# Supplementary material for: Identification of Patients with Potential Atrial Fibrillation during Sinus Rhythm Using Isolated P Wave Characteristics from 12-Lead ECGs
Source: J Pers Med. 2022 Sep 29;12(10):1608. doi: 10.3390/jpm12101608 (PMC9604932; doi:10.3390/jpm12101608)

Supplementary Materials:

Figure S1. CNN architecture used in our study.

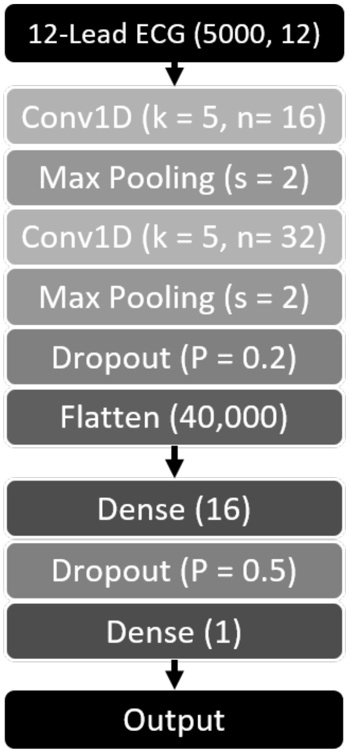

Figure S2. SHAP summary plot showing the relationship between the value of each feature and the impact on AF probability.

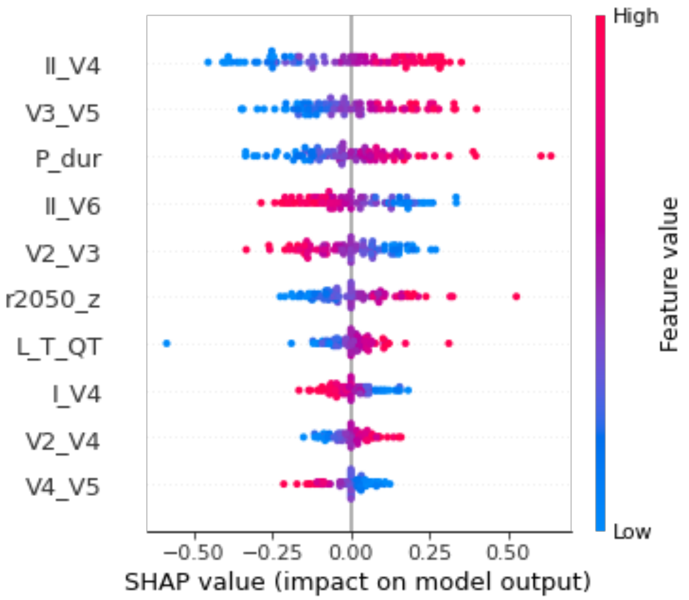

Supplement: Supplementary file 1 [file jpm-12-01608-s001.zip › jpm-1796577-supplementary.pdf]
